# From Accumulation to Degradation: Reprogramming Polyamine Metabolism Facilitates Dark-Induced Senescence in Barley Leaf Cells

**DOI:** 10.3389/fpls.2015.01198

**Published:** 2016-01-06

**Authors:** Ewa Sobieszczuk-Nowicka, Szymon Kubala, Agnieszka Zmienko, Arleta Małecka, Jolanta Legocka

**Affiliations:** ^1^Department of Plant Physiology, Faculty of Biology, Adam Mickiewicz University in PoznańPoznań, Poland; ^2^Laboratory of Molecular and Systems Biology, Institute of Bioorganic Chemistry – Polish Academy of SciencesPoznań, Poland; ^3^Institute of Computing Science, Poznań University of TechnologyPoznań, Poland; ^4^Department of Biochemistry, Faculty of Biology, Adam Mickiewicz University in PoznańPoznań, Poland

**Keywords:** barley, leaf, metabolism, polyamines, senescence, transcriptional profiling

## Abstract

The aim of this study was to analyze whether polyamine (PA) metabolism is involved in dark-induced *Hordeum vulgare* L. ‘Nagrad’ leaf senescence. In the cell, the titer of PAs is relatively constant and is carefully controlled. Senescence-dependent increases in the titer of the free PAs putrescine, spermidine, and spermine occurred when the process was induced, accompanied by the formation of putrescine conjugates. The addition of the anti-senescing agent cytokinin, which delays senescence, to dark-incubated leaves slowed the senescence-dependent PA accumulation. A feature of the senescence process was initial accumulation of PAs at the beginning of the process and their subsequent decrease during the later stages. Indeed, the process was accompanied by both enhanced expression of PA biosynthesis and catabolism genes and an increase in the activity of enzymes involved in the two metabolic pathways. To confirm whether the capacity of the plant to control senescence might be linked to PA, chlorophyll fluorescence parameters, and leaf nitrogen status in senescing barley leaves were measured after PA catabolism inhibition and exogenously applied γ-aminobutyric acid (GABA). The results obtained by blocking putrescine oxidation showed that the senescence process was accelerated. However, when the inhibitor was applied together with GABA, senescence continued without disruption. On the other hand, inhibition of spermidine and spermine oxidation delayed the process. It could be concluded that in dark-induced leaf senescence, the initial accumulation of PAs leads to facilitating their catabolism. Putrescine supports senescence through GABA production and spermidine/spermine supports senescence-dependent degradation processes, is verified by H_2_O_2_ generation.

## Introduction

In plants and animals, developmental and growth processes require selective elimination of either single cells or groups of cells. This process, termed PCD, is actively controlled by the organism. In plants, PCD may involve the elimination of an entire organ; e.g., a leaf that for many reasons no longer has a useful role. When senescence occurs, it is not a steady state but a gradual evolution of the entire cell, even though it can sometimes be delayed or reversed, preceding cell death (Cai et al., 2015).

Polyamines are multi-functional polycationic compounds found in plants, animals, fungi, and bacteria. PA research in plants has been mainly focused on three aliphatic amines: putrescine, spermidine, and SM/T-SM (Takahashi and Kakehi, 2010). In plants, PAs are involved in many physiological and developmental processes. Their roles in growth, metabolism, stress tolerance, and crosstalk with phytohormones or ion channels and pumps has been recently described in an excellent book edited by Kusano and Suzuki (2015). Numerous studies have also linked PAs to the regulation of plant cell senescence, although the information regarding this aspect is still fragmentary. PAs have been implicated in the prolonged survival of excised organs or senescent organs *in vivo*, such as leaves, flowers, and fruits. However, some contradictory reports discuss whether the PA level increases or decreases during senescence (Legocka and Zajchert, 1999; Lester, 2000; Serafini-Fracassini et al., 2002, 2010; Seiler and Raul, 2005; Bagni and Tassoni, 2006; Kusano et al., 2008; Nambeesan et al., 2010). In one of the first reports on the role of PAs in senescing leaves, Cohen et al. (1979) stated that PAs prevented the loss of chlorophyll normally associated with the senescence of excised leaves maintained in the dark. They also suggested that the influence of PAs on senescence-related processes might be due to their cationic nature, which enables direct interaction with nucleic acids, phospholipids and many proteins. Mizrahi et al. (1989) reported that PAs delayed leaf senescence in oats and petunias, and noticed a strong association of PAs and proteins of high molecular weight, suggesting that this protein-bound PA fraction might be involved in the observed changes. PAs were also found to play an important role in delaying chloroplast degradation in oat leaves exposed to osmotic stress (Besford et al., 1993). Likewise, the addition of SD or SM inhibited protein degradation and chlorophyll loss, and stabilized thylakoid proteins such as D1, D2, cyt *f* and the large subunit of RuBisCO (Besford et al., 1993; Legocka and Zajchert, 1999; Serafini-Fracassini et al., 2010).

Most studies on the role of PA in plant senescence (Cohen et al., 1979; Mizrahi et al., 1989; Besford et al., 1993; Legocka and Zajchert, 1999; Mattoo et al., 2006; Mattoo and Handa, 2008; Nambeesan et al., 2010; Serafini-Fracassini et al., 2010) investigated the effect of exogenously applied PAs or overproduced PAs. Another common approach is measuring the concentration of free PAs in tissue extracts, but this provides only a “snapshot” picture of a continuously changing environment, as the cellular levels of PAs reflect the balance of their synthesis, catabolism, attachment to other molecules and transport (Kusano and Suzuki, 2015). Very recently, Ioannidis et al. (2014) demonstrated that the mRNA for DAO, PAO were up-regulated during leaf aging. This finding indicated that the internal PA pool undergoes regulation in senescing plant cells. It is not known, however, whether and how PA metabolism is linked to the sequence of physiological changes that ultimately lead to cell death. In particular, it is not evident whether PAs act as mediators in this process. Does the selective change in the level of the free, conjugated or bound form of PAs regulate the leaf senescence? What mechanisms actually cause the increase or decrease in PA titer during senescence, and which signaling molecules participate in this response? If PAs indeed control the senescence progress, is a particular PA responsible? Is PA synthesis and/or catabolism affected and does any senescence-dependent crosstalk between different branches of PA metabolism occur? With all these questions in mind, we decided to investigate whether the ability of plants to control senescence is related to their capacity to metabolize PAs. Recently, we reported on changes in the activity of transglutaminase and in the level of PAs bound to thylakoids in an experimental model, in which the senescence in barley leaves was dark-induced (Sobieszczuk-Nowicka et al., 2009, 2015). Here, we extend our studies on the fate of internal PAs in this model by evaluating the levels of PU, SP, SM, and DP in distinct PA fractions (free, conjugated, and apoplastic PAs) and measuring the transcript levels and protein activity of genes involved in PA synthesis and catabolism during 10 days of barley leaf senescence. We also put the observed changes in the broader physiological context by following in parallel the changes in the accumulation of reactive oxygen species (H_2_O_2_), chloroplast decomposition, and effect of hormone (cytokinin) treatment, as well as the expression of genes related to carbon and nitrogen metabolism and ethylene biosynthesis. This multidisciplinary approach contributes to a better understanding of the role of PA in controlled plant cell death. Additionally, the study of the senescence process in the monocotyledonous crop plant (barley) is an important issue in relation to the crop yield because leaf senescence, triggered by many types of environmental stress, is unfavorable for the agriculture industry.

## Materials and Methods

### Plant Material and Treatments

Barley (*Hordeum vulgare* L. ‘Nagrad’) seedlings were grown for 7 days on soil under controlled conditions (day/night 16/8 h, 23°C, light intensity 150 μmol m^-2^ s^-1^, 60% humidity). The material for the day 0 sample was then collected. Then, primary leaves were detached and their bases were placed in water (control), 400 μM KIN, 10 μM AG, 50 μM guazatine (G) or 10 μM AG and 1 mM GABA. The senescence process was initiated by incubation in the dark and samples were collected after 3, 7, and 10 days. In detached leaves, PAs are free to eﬄux in the water or medium, which may potentially decrease the endogenous titer of the PAs. We initially compared the free PA levels in the detached and intact leaves subjected to darkness-induced senescence. In our model, leaf detachment had little influence on observed PA level.

### Isolation and Quantification of PA Fractions

To obtain distinct PA fractions, leaves were first powdered with liquid nitrogen and homogenized with 5% perchloric acid (PCA, 100 mg ml^-1^). The homogenates were centrifuged at 27,000 × *g* for 20 min and the supernatant was collected (free PAs fraction). Part of the supernatant was mixed in a 1:1 ratio with 12 M HCl and heated at 110°C for 16 h. The resulting mixture was filtered and dried. Then, the dried material was dissolved in 5% PCA. The hydrolyzed PCA supernatant contained the PAs liberated from the PCA-soluble conjugate fraction. The apoplastic fluid PA fraction was isolated and analyzed as previously described (Yoda et al., 2003, 2006; Moschou et al., 2008). All PA fractions were then dansylated with dansyl chloride. Dansylated PAs were collected with toluene and, after toluene evaporation, dissolved in 800 μl acetonitrile. Quantitative and qualitative analyses of the PAs PU, SD, SM, and DP were performed by an HPLC method according to Marcé et al. (1995). Detailed information about sample application, column, flow rate, gradient elution, and retention times of the different PAs was developed as described by Sobieszczuk-Nowicka et al. (2015).

### Microarray Data Analysis

The microarray experiment surveying gene expression in intact senescing barley leaves after induction by darkness has been described by Zmienko et al. (2015a,b). Gene expression is presented based on the oligonucleotide probe data as log2-fold changes relative to day 0. MapMan analysis was performed with the entire microarray dataset based on the Agilent probe mapping files for barley downloaded from the MapMan repository. For genes related to PA metabolism and associated metabolic pathways that were not identified based on MapMan analysis, barley homologs were searched with the BLAST + tool using amino acid sequences of well-described genes from other plant species. Whenever a barley homolog was found, its sequence was used to search for Agilent oligonucleotide probes present on the microarray in the sense orientation with the following filters: -evalue 0.0001 -word_size 7 -outfmt 1 -dust no -perc_identity 65.

### Enzyme Activity Assays

Arginine decarboxylase and SAMDC activities were determined by radiochemical methods as described by Legocka and Żarnowska (2000) and Sobieszczuk-Nowicka et al. (2007), respectively, with minor modifications described below. Plant material was homogenized in 100 mM Tris-HCl (pH 7.6) containing 25 μM pyridoxal phosphate and 50 μM EDTA (200 mg ml^-1^). Samples were centrifuged at 20,000 × *g* for 30 min at 4°C. The supernatant was used to assay ADC or SAMDC activity by measuring counts per minute (CPM) of [^14^CO_2_] evolution from 1.48 kBq L-[l-^14^C] arginine (the specific activity of L-[l-^14^C] arginine was 11.8 GBq mmol^-1^) or 3.7 kBq [1-^14^C]-*S*-adenosyl-L-[carboxyl-^14^C]methionine (the specific activity of *S*-adenosyl-L-[carboxyl-^14^C] methionine was 2.04 GBq mmol^-1^) per mg protein. The protein content of the thylakoid-enriched fraction was determined using the bicinchoninic acid method (Brown et al., 1989).

Diamine oxidase and PAO crude enzyme extracts were obtained according to the protocol described by Xing et al. (2007) and used to determine DAO and PAO activity. The reaction solutions (3.0 ml) contained 2.5 ml 0.1 M sodium phosphate buffer (pH 6.5), 0.1 ml crude enzyme extracts/apoplastic fluids, 0.1 ml peroxidase (250 U ml^-1^) and 0.2 ml 4-aminoantipyrine/*N,N*-dimethylaniline (0.5 mM 4-aminoantipyrine and 0.025% *N,N*-dimethylaniline in 0.1 M phosphate buffer). The reaction was initiated by the addition of 0.1 ml of 20 mM PU or 20 mM SD, respectively. A 0.01 value of the changes in absorbance at 555 nm was regarded as one activity unit of the enzyme.

### Measurement of Chlorophyll *a* Fluorescence Induction Kinetics

*Chl a* fluorescence was measured at room temperature with an FSM 1 fluorometer (Hansatech) run by Modfluor software. The fluorometer was connected to a leaf-clip holder through a fiber optic cable. Prior to each measurement, the leaves were dark-adapted for 20 min. The procedure of Jackowski et al. (2003) was followed. The minimal fluorescence level (*F_0_*) was determined in dark-adapted leaves using the modulated beam. The maximal fluorescence level (*F_m_*) was determined by a 0.3 s saturating pulse. Then, the leaves were continuously illuminated with a white actinic light at an irradiance level equivalent to the one used for acclimatization (150 μmol m^-2^ s^-1^) until the steady-state value of fluorescence (*F_s_*) was reached after 1–2 min. A second saturating pulse was then imposed to determine the maximal fluorescence level in the light-adapted state (*F*’_m_). The actinic light was then turned off and the minimal fluorescence level in the light-adapted state (*F*’_0_) was established after a 3 s illumination of the leaf with a far-red beam.

The *Chl* fluorescence kinetic parameters were calculated as:

Maximum quantum yield of PSII in the dark adapted state,

$$F\,v/F\,m=\left(F\,m-F_{0}\right)/F\,m$$

Photochemical quenching of *Chl* fluorescence,

$$q\,P=F\,m^{,}-F/F\,m^{,}-F_{0}$$

Non-photochemical quenching of *Chl* fluorescence,

$$N\,P\,Q=\left(F\,m-F\,m^{,}\right)/F\,m^{,}$$

### Estimation of Plant Nitrogen Status

The method based on fluorometric measurement of the chlorophyll/flavonoids ratio with the use of a DUALEX 4 FLAV (Force-A, Orsay, France) fluorometer was employed (Cartelat et al., 2005). The amount of *Flv* was estimated from the difference in *Chl* fluorescence induced by UV and red light, since a portion of UV light is absorbed by *Flv.* The *Chl* level was measured by leaf red light transmittance, corrected for transmittance caused by other leaf structures.

### *In vivo* Detection of Hydrogen Peroxide

Senescing barley leaves were submerged for 12 h in 4 μM dichlorodihydrofluorescein diacetate (DCFH-DA) in 5 mM dimethyl sulfoxide according to Afzal et al. (2003). They were then washed twice with 50 mM phosphate buffer (pH 7.4) and the leaves were observed with a confocal microscope (the Zeiss LSM 510 model, Axioverd 200 M, Jena, Germany) with a filter set no. 10, excitation 475 nm, and emission 520 nm or higher.

### Spectrophotometric Determination of Hydrogen Peroxide

Spectrophotometric determination of hydrogen peroxide (H_2_O_2_) based on titanium (Ti^4+^) was performed according to Becana et al. (1986). Barley leaves (500 mg) were homogenized in 6 ml of 100 mM phosphate buffer, pH 7.8. The homogenate was centrifuged at 15,000 *g* for 30 min at 4°C. For spectrophotometric measurement, the reaction mixture contained 100 mM phosphate buffer, pH 7.8, plant extract and the titanium reagent consisting of 0.3 mM 4-(2-pyridylazo)resorcinol and 0.3 mM titanium potassium titrate in the ratio 1:1. Absorbance was measured at λ = 508 nm against a calibration curve prepared for the content of H_2_O_2_ from 0 to 100 nM.

### Statistical Analysis

The differences in the measured parameters were analyzed for statistical significance using one-way analysis of variance (ANOVA) and the Tukey–Kramer Multiple Comparison Test. Means were considered as significantly different at a *p*-value < 0.01. Differential gene expression was evaluated using Bayesian linear modeling (R/Bioconductor, limma package) with Benjamini and Hochberg’s correction of the false discovery rate. The adjusted *p*-values are indicated as ^∗^*p* < 0.05, ^∗∗^*p* < 0.005, and ^∗∗∗^*p* < 0.0005.

## Results

### Changes in the Level of Different PA Fractions during the Senescence of Detached Barley Leaves

**FIGURE 1:**
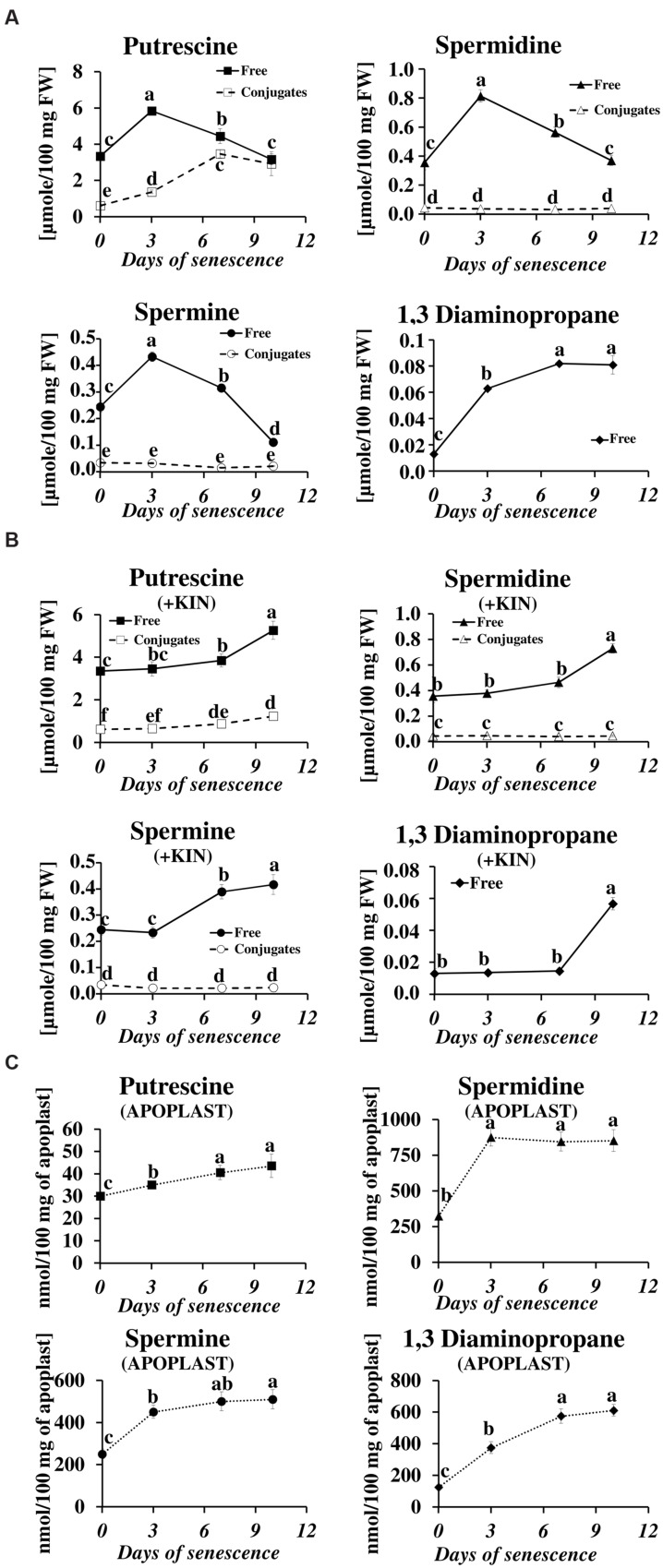
**Senescence-related changes in free, conjugated and apoplastic fluid polyamine levels in dark-incubated barley leaves. (A)** Free (solid line) and conjugated (dashed line) polyamines. **(B)** Free (solid line) and conjugated (dashed line) polyamines in leaves additionally treated with exogenous 400 μM kinetin (KIN). **(C)** Apoplastic fluid polyamines. Putrescine, spermidine, spermine, and diaminopropane concentrations were determined through senescence using HPLC after polyamine derivatization with dansyl chloride. The values are reported in micromoles relative to leaf fresh weight (FW). The differences in the measured parameters were analyzed for statistical significance using one-way analysis of variance (ANOVA) and the Tukey–Kramer Multiple Comparison Test (*n* = 9, *p* < 0.01). The same letters on bars describe non-significant differences between means.

We have studied the process of senescence of barley leaves using a dark-induction model that we routinely use in our research (Legocka and Zajchert, 1999; Sobieszczuk-Nowicka et al., 2009, 2015). Dark-induced barley leaf senescence is a dynamic process leading through a series of transformations to the decay of the photosynthetic apparatus, which in turn decreases photosynthetic capacity of the leaf, the disruption of the organelles and ultimately to cell death. Our recent observations regarding symptoms of chlorophyll loss in senescing leaves, decomposition of chloroplasts, internucleosomal fragmentation of chromatin, condensation of nuclear DNA and the disruption of the nucleus indicate that dark-induced leaf senescence engages PCD mechanisms (Zmienko et al., 2015a). To verify whether this process also involves changes in PA titer, we determined levels of free and conjugated PAs (PU, SD, SM, and DP) in the detached leaves throughout the process (**Figure 1A**). The PU level increased to a maximum (up to 1.74× of the level observed at the onset of senescence induction) at day 3 of senescence. At day 7 it slightly decreased and finally returned to the initial level at day 10. The relative changes in level of free SD mimicked those of PU, with a maximum increase (2.3×) at day 3, followed by a slight decrease at day 7 and returning to the initial level at day 10. Likewise, the amount of free SM peaked at day 3 (1.8 times increase) but decreased faster and dropped 2.2 times below the initial level at day 10. The amount of free DP showed a stepwise increase throughout the entire period of leaf senescence. At day 10 it was 6.2× higher in comparison to day 0. However, the percentage of DP in the total pool of free PAs was negligible. Accumulation of free PU was accompanied by increased formation of PU conjugates (**Figure 1A**). Their level increased fivefold between days 0 and 10. No other conjugates, either SD or SM, accumulated.

The changes in the levels of free and conjugated PAs during senescence were also evaluated after the addition of the anti-senescing agent KIN. Exogenously added KIN retards the senescence process (Sobieszczuk-Nowicka et al., 2009 and references therein). Here, KIN slowed the accumulation of all free PAs in the detached leaves. In those incubated with KIN, high levels of free PU, SD, SM, and DP (comparable to their amounts at day 3 without KIN addition, see **Figure 1A**) were reached only at days 7–10 (**Figure 1B**). The accumulation of PU conjugates was similarly delayed by the addition of KIN.

In the apoplastic fluids, PA levels also increased during the senescence process, but the ratios of individual PAs and their accumulation profiles were different compared to the free PA fraction (**Figure 1C**). PU, which was the most abundant of the free PAs (**Figure 1A**), was one order of magnitude less abundant than the other PAs (**Figure 1C**) in the apoplastic fluids pool. It displayed a slight but continuous increase from 30 nmol/100 mg apoplast at day 0 to 43.5 nmol/100 mg apoplast at day 10. The amounts of SD and SM increased substantially at day 3 (from 324 to 875 nmol/100 mg apoplast for SD and from 250 to 450 nmol/100 mg apoplast for SM) and remained approximately at that level (SD) or slightly higher (SM) at days 7 and 10. DP levels increased continuously through the entire time of senescence, accumulating from 125 to 612 nmol/100 mg apoplast, with the most rapid changes observed at days 3 and 7 and a slower increase at day 10.

### Senescence-Associated Changes in the Transcript Levels and Protein Activity of Genes Directly Involved in PA Metabolism

**FIGURE 2:**
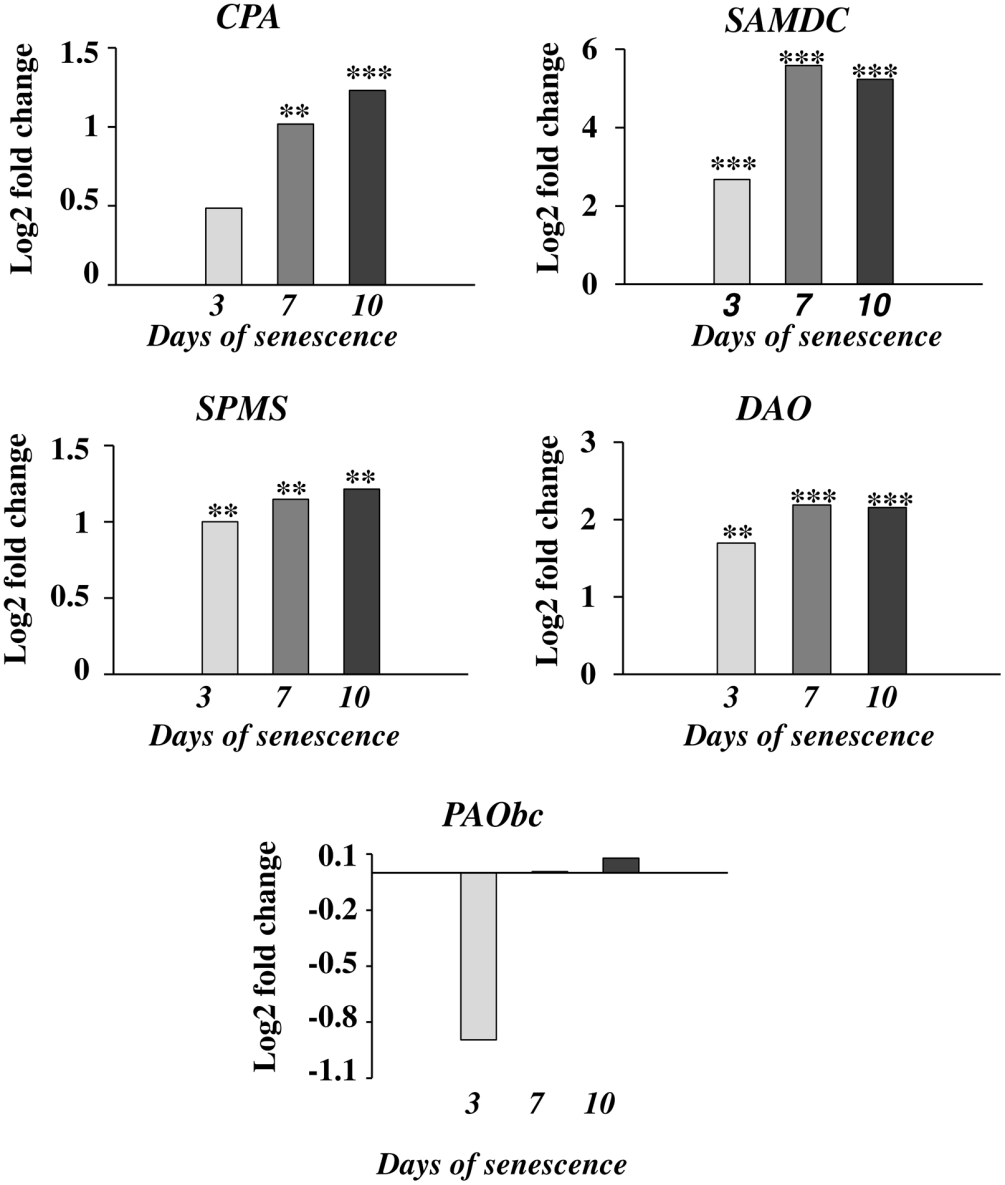
**The time course of *CPA, SAMDC, SPMS*, *DAO*, and *PAObc* expression in barley leaves undergoing dark-induced senescence**. CPA, *N*-carbamoylputrescine amidohydrolase; SAMDC, SAM decarboxylase; SPMS, spermine synthase; DAO, diamine oxidase; PAObc, back conversion polyamine oxidase. Barley homologs of each gene were found by homology searches in public databases. The gene expression data are from a time-course experiment performed with an Agilent oligonucleotide microarray (Zmienko et al., 2015a,b). The data are presented as log2-fold changes relative to day 0. Differential gene expression was evaluated with Bayesian linear modeling (R/Bioconductor, limma package) with Benjamini and Hochberg’s correction of the false discovery rate. The adjusted *p*-values are marked with ^∗^*p* < 0.05, ^∗∗^*p* < 0.005, and ^∗∗∗^*p* < 0.0005.

The accumulation of various fractions of PAs observed in the barley leaves undergoing senescence might result from the increased synthesis or activation of proteins involved in PA production and also from other mechanisms; e.g., PA transport. We first investigated whether changes in PA levels were accompanied by any changes in the expression of genes involved in PA metabolism. We utilized previously generated microarray data used to evaluate barley gene expression in the same experimental model of dark-induced senescence, except that the leaves were not detached (Zmienko et al., 2015a,b). Regarding barley genes involved in PA metabolism, only *SPMS* and two *PAO* genes were characterized in detail (Cervelli et al., 2006; Rodriguez-Kessler and Jiménez-Bremont, 2008). For this reason, we performed homology-based searches in public databases using the sequences of PA metabolism genes from other species to identify barley homologs. We then examined gene expression during senescence based on the above microarray datasets (**Figure 2**). With this approach, up-regulation of four genes was detected. A gene encoding *CPA* was induced 1.4-4-fold; a gene encoding *DAO* was induced 1.6-2.1-fold; and a gene encoding *SPMS* was induced 3.5-4-fold. The most remarkable changes were detected in the expression of the *SAMDC* gene, reaching ninefold induction at day 3 and 18.5-17.5-fold induction at later stages of senescence of leaves. On the other hand, the changes in expression level of the gene encoding *PAObc* were transient and not statistically significant.

**FIGURE 3:**
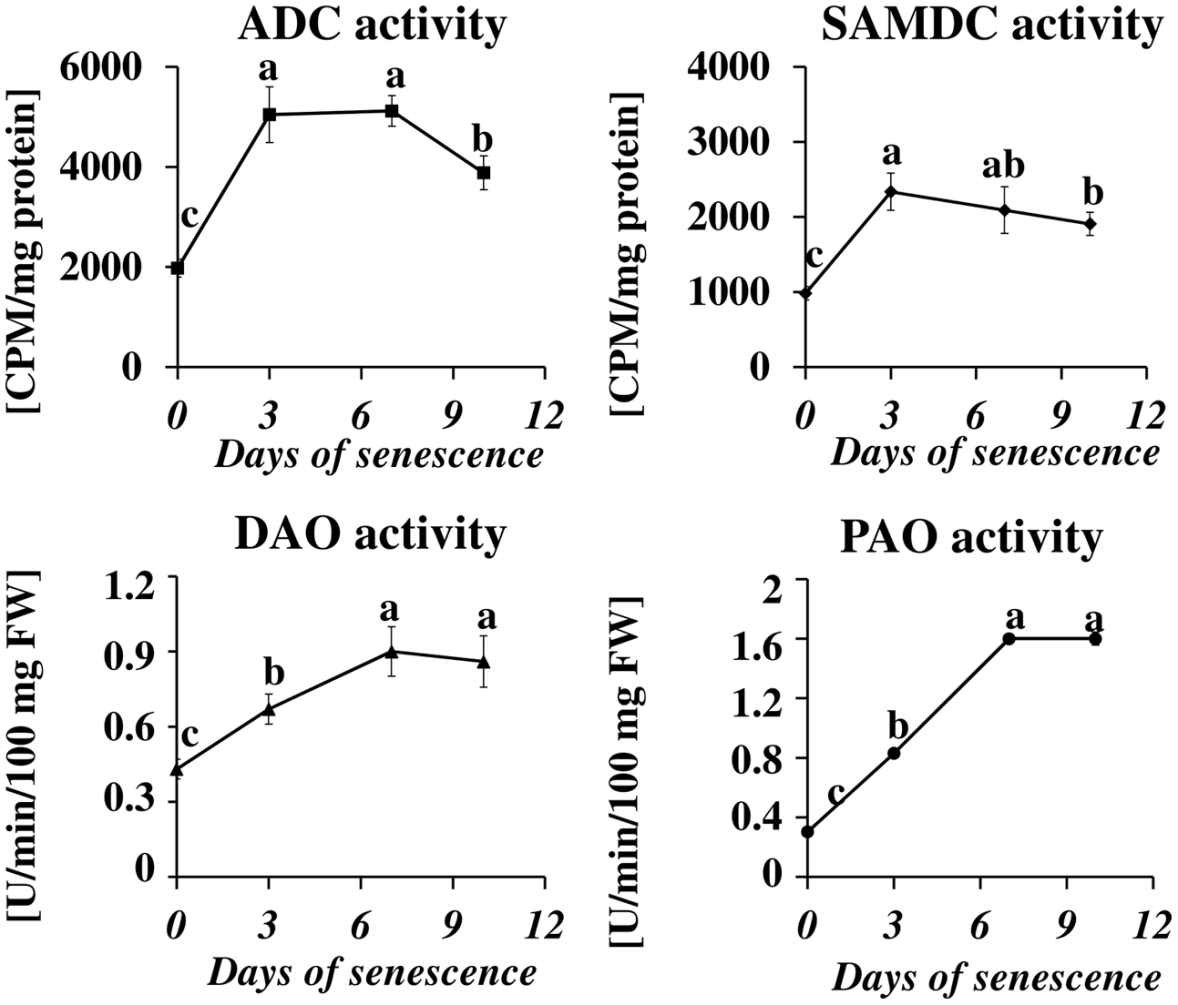
**The time course of ADC, SAMDC, DAO, and PAO activities in barley leaves undergoing dark-induced senescence**. ADC, arginine decarboxylase, SAMDC, *S*-adenosyl-methionine decarboxylase; DAO, diamine oxidase; PAO, polyamine oxidase. Enzyme activities were determined as described in Section “Materials and Methods.” The values for ADC and SAMDC activity were reported in CPM relative to protein in mg. DAO and PAO activity are reported in U relative to time (min) and leaf fresh weight (FW). The differences in the measured parameters were analyzed for statistical significance using one-way ANOVA and the Tukey–Kramer Multiple Comparison Test (*n* = 6, *p* < 0.01). The same letters on bars describe non-significant differences between means.

Next, we measured the senescence-associated changes in the activity of proteins engaged in PA metabolism: ADC, SAMDC, DAO, and PAO (**Figure 3**). A rapid increase in the activity of both enzymes involved in PA biosynthesis (SAMDC and ADC) was observed at day 3 (2.4× and 2.6×, respectively), followed by a slight decline at days 7 and 10 but still well-above the initial level. The activity of enzymes engaged in PA catabolism (DAO and PAO) continuously increased to day 7, when it reached maximal values (2× and 5.3×, respectively) and stabilized at that level.

### Senescence-Associated Changes in Transcript Levels of Genes Involved in Pathways Related to PA Metabolism

**FIGURE 4:**
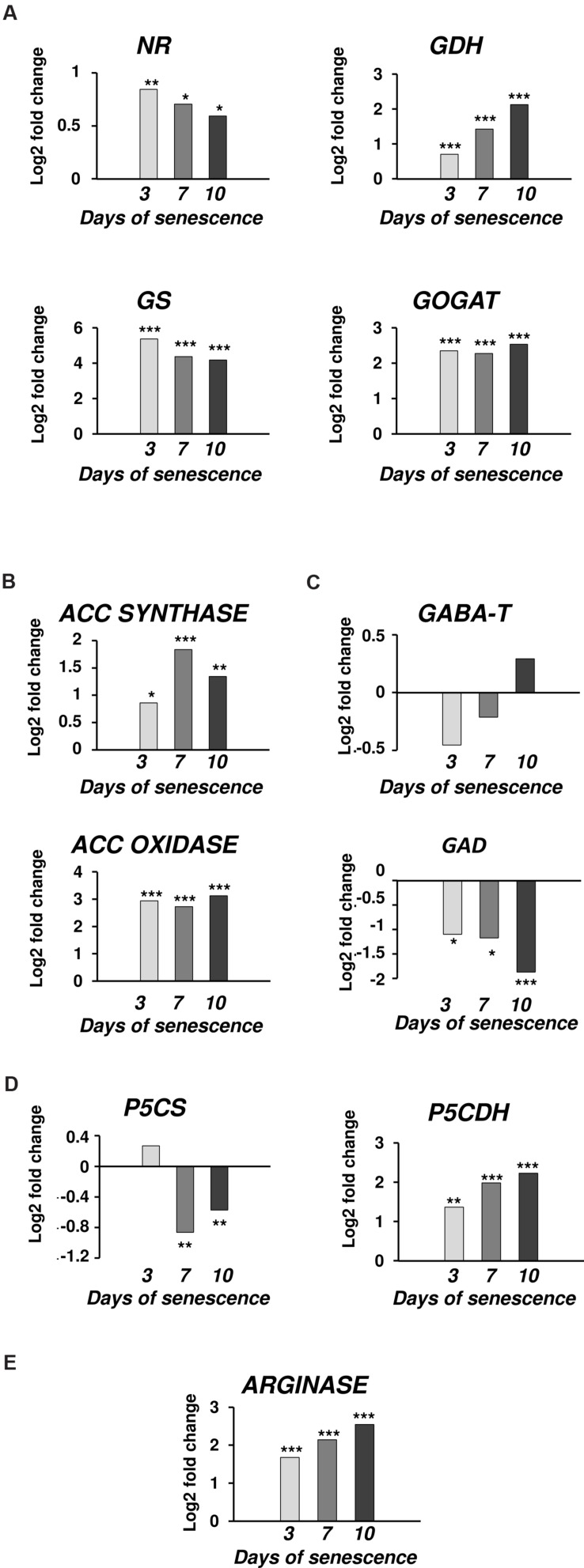
**The time course of *NR, GDH, GS, GOGAT, ACC SYNTHASE, ACC OXIDASE, GABA-T, GAD, P5CS, P5CDH*, and *ARGINASE* expression in barley leaves undergoing dark-induced senescence. (A)** genes involved in nitrate metabolism; NR, nitrate reductase; GDH, glutamate dehydrogenase; GS, glutamine synthetase; GOGAT, glutamine oxoglutarate aminotransferase. **(B)** genes involved in ethylene synthesis; *ACC SYNTHASE; ACC OXIDASE.* **(C)** Genes involved in GABA metabolism; GABA-T, γ-aminobutyrate aminotransferase; GAD, glutamate decarboxylase. **(D)** Genes involved in proline metabolism; P5CS, Δ^1^ pyrroline-5-carboxylate synthetase; P5CDH, Δ^1^ pyrroline-5-carboxylate dehydrogenase. **(E)** Gene involved in urea metabolism; *ARGINASE*. Barley homologs for each gene were found by homology searches in public databases. The gene expression data are from the time-course experiment performed with the Agilent oligonucleotide microarray (Zmienko et al., 2015a,b). The data are presented as log2-fold changes relative to day 0. Differential gene expression was evaluated with Bayesian linear modeling (R/Bioconductor, limma package) with Benjamini and Hochberg’s correction of the false discovery rate. The adjusted *p*-values are marked with ^∗^*p* < 0.05, ^∗∗^*p* < 0.005, and ^∗∗∗^*p* < 0.0005.

The microarray data for dark-induced senescing barley leaves allowed us to also examine the transcriptional profiles of genes encoding enzymes from other metabolic pathways that are strictly related to PA synthesis and turnover (**Figure 4**). Several genes involved in nitrate metabolism were represented by adequate oligonucleotide probes on the microarray. All showed an increased expression from day 3 throughout the whole period of leaf senescence (**Figure 4A**). The expression of the *GOGAT* was constantly up-regulated, approximately fivefold. The gene encoding *GDH* was progressively up-regulated, reaching a 4.3-fold increase at day 10. The expression of the gene encoding *GS* initially increased 38× and then slightly dropped, but still remained much higher than at day 0. A similar trend was observed for the *NR* gene, except its induction was much lower (maximum 1.8× at day 3). Two genes involved in ethylene metabolism and represented on the microarray also were up-regulated: *ACC synthase*, up to 3.5-fold at day 7, and *ACC oxidase*, up to 8.7-fold at day 10 (**Figure 4B**). Two genes involved in GABA metabolism (**Figure 4C**) and two involved in proline metabolism (**Figure 4D**) were also investigated. While the gene encoding *GAD* was down-regulated during the entire period, with the strongest reduction in expression level at day 10 (3.6-fold), the expression of the gene encoding *GABA-T* did not change by more than 50% during the entire period of leaf senescence (**Figure 4C**). *P5CS* was up-regulated at day 3 (1.2-fold) and then was down-regulated with the strongest reduction in expression level at day 7 (0.55-fold), whereas *P5CDH* was up-regulated during the entire period, with the strongest increase in expression level at day 10 (4.7-fold; **Figure 4D**).The *arginase* gene, related to urea metabolism, was progressively up-regulated, with a maximum increase of 5.8-fold at day 10 (**Figure 4E**).

### Effect of PA Catabolism Inhibition on Senescence-Related Changes in Free Polyamine Titer

**FIGURE 5:**
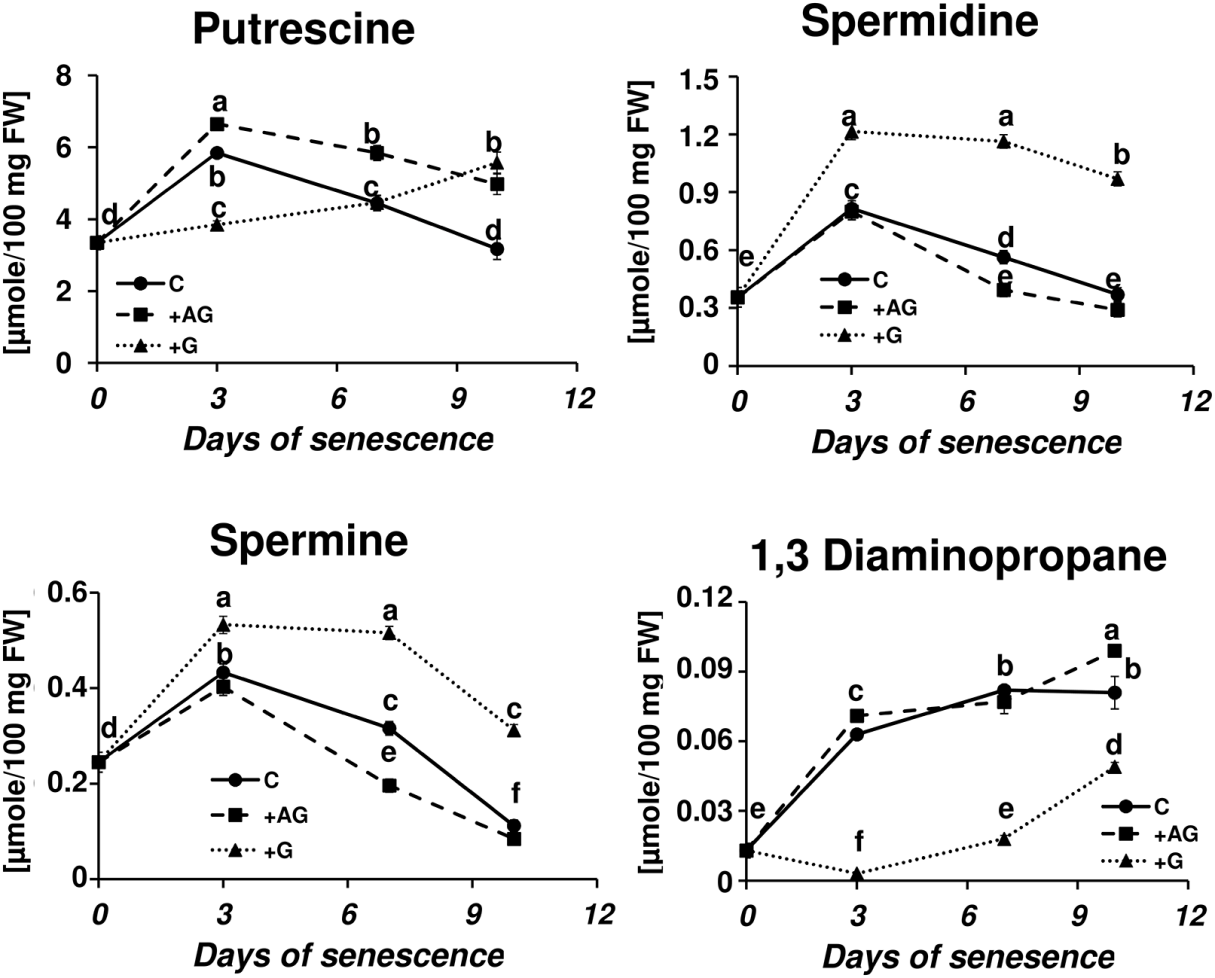
**Senescence-related changes in free polyamine level in dark-incubated conditions in the presence of PA catabolism inhibitors in barley leaves**. Polyamines (PAs) were estimated in C (solid line)- control leaves and after application of polyamine catabolism inhibitors: +AG (dashed line) – leaves treated with 10 μM *N,N*-diaminoguanidine (AG) – diamine oxidase (DAO) inhibitor; +G (dotted line) leaves treated with 50 μM guazatine (G) – polyamine oxidase (PAO) inhibitor. Putrescine, spermidine, spermine, and diaminopropane concentrations were determined using HPLC after polyamine derivatization with dansyl chloride. The values are reported in micromoles relative to leaf FW. The differences in the measured parameters were analyzed for statistical significance using one-way ANOVA and the Tukey–Kramer Multiple Comparison Test (*n* = 12, *p* < 0.01). The same letters on bars describe non-significant differences between means.

Transcriptional profiling results revealed that PA catabolism might be an important element regulating dark-induced leaf senescence, which encouraged us to evaluate the effect of inhibiting that pathway. The two inhibitors used in the studies were AG, a competitive inhibitor of soybean DAO (Nikolov et al., 1990; Xing et al., 2007), and guazatine (G), which competitively inhibits maize and tobacco PAOs (Cona et al., 2004; Yoda et al., 2006). The efficiency of inhibition of the enzyme activities was evaluated after treatment with each inhibitor by measuring the respective oxidase activity compared to the control (C – detached leaves without inhibitor treatment). AG inhibited DAO activity by 69% at day 3 and 58% at days 7–10. G inhibited PAO activity by 90% at day 3 and approximately 75% at days 7–10. We next looked at the level of free PAs in inhibitor-treated senescing leaves (**Figure 5**). The treatment with AG induced a PU level higher than in the control plants (1.13×, 1.3×, and 1.56× at days 3, 7, and 10, respectively), while the accumulation of SD and SM during senescence was slightly lower, with the differences most observable at day 7, when their levels reached 0.7 and 0.62 of the control amount, respectively. The DP level did not differ much from the control at any time point (maximum observed difference was a 1.2× increase at day 10). The inhibition of PAO activity delayed the accumulation of PU; its level slowly increased and at day 10 it reached 5.575 μmol/100 mg FW, comparable with the amount observed in the control at day 3. Treatment with G also drastically lowered DP accumulation. Its level was even lower at day 3 than at day 0 and gradually increased afterward, but even by day 10 it did not reach the level that was observed in the control plants at day 3. On the contrary, PAO activity inhibition caused overall elevation of SD and SM levels compared to the control, but did not alter the accumulation speed. The SD level in leaves treated with G was 1.5×, 2.06×, and 1.7 higher at days 3, 7, and 10, respectively, than in the control. The SM level in leaves treated with G was 1.2×, 1.6×, and 2.78 higher at days 3, 7, and 10, respectively, than in the control.

### Effect of PA Catabolism Inhibition on Photosynthesis in Senescing Barley Leaves

**FIGURE 6:**
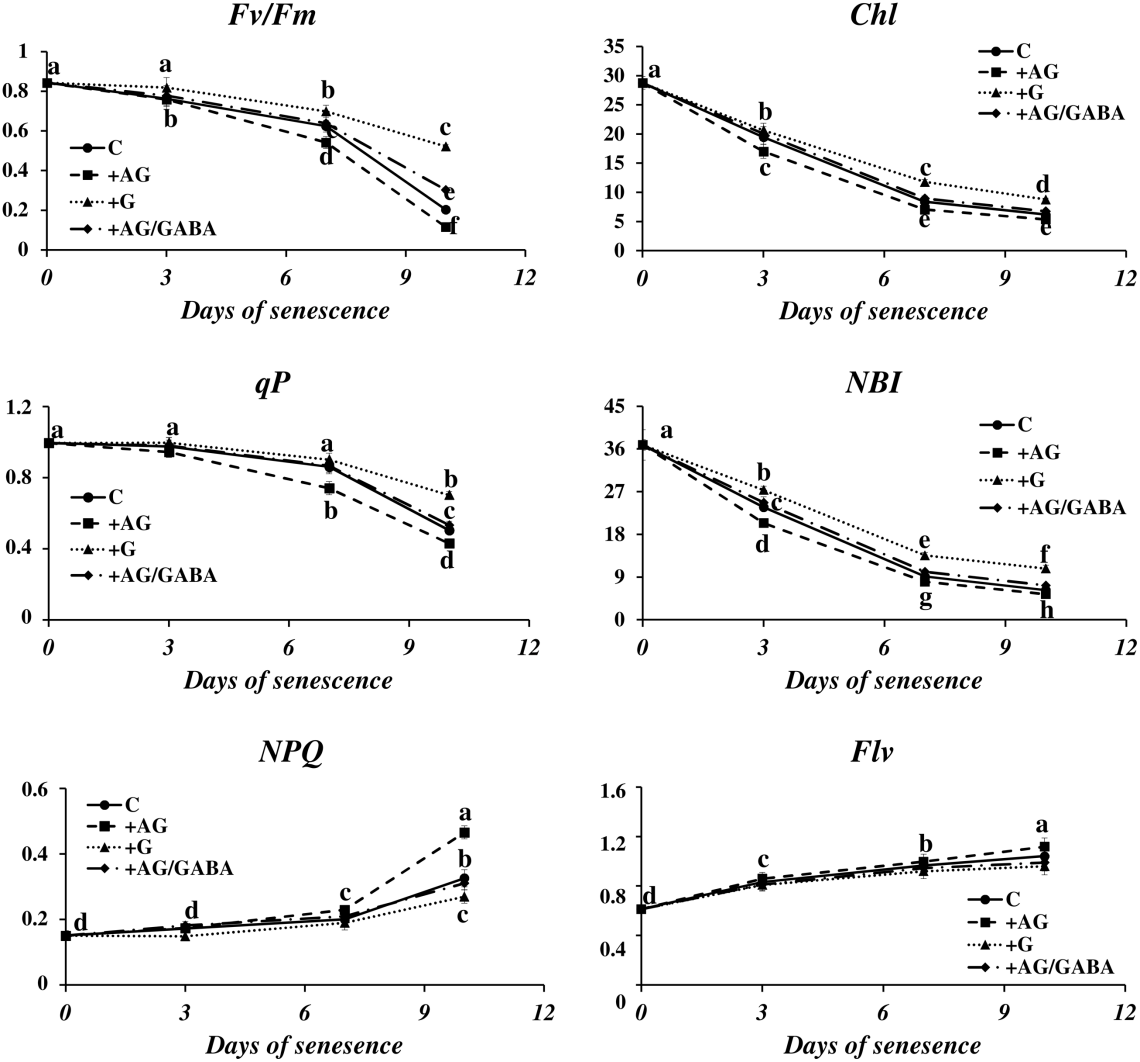
**Senescence-related changes in chlorophyll fluorescence and nitrogen status in dark-incubated conditions in the presence of PA catabolism inhibitors in barley leaves**. The parameters *Fv*/*Fm*, maximum quantum yield of PSII in the dark adapted state; *qP*, photochemical quenching *Chl* fluorescence; *NPQ*, non-photochemical quenching of *Chl* fluorescence; *Chl*, chlorophyll level; *NBI*, nitrogen balance index; and *Flv*, amount of flavonoids were measured throughout senescence in C (solid line)- control leaves and after application of polyamine catabolism inhibitors: + AG (dashed line) – leaves treated with 10 μM *N,N*-diaminoguanidine (AG) – diamine oxidase (DAO) inhibitor; + AG/GABA (dashed-dotted line) – leaves treated with 10 μM AG and 1 mM γ-aminobutyric acid (GABA);+ G (dotted line) leaves treated with 50 μM guazatine (G) – polyamine oxidase (PAO) inhibitor. Measurements of *Chl* fluorescence induction kinetics were taken using a pulse amplitude-modulated PAM fluorimeter. For estimation of plant nitrogen status, a method based on fluorometric measurement of chlorophyll/flavonoids ratio with a DUALEX 4 FLAV fluorimeter was used. The differences in the measured parameters were analyzed for statistical significance using one-way ANOVA and the Tukey–Kramer Multiple Comparison Test (*n* = 25, *p* < 0.01). The same letters on bars describe non-significant differences between means.

The disintegration of chloroplasts and cessation of photosynthesis is an important landmark of the leaf senescence process, because the dying process of a plant cell may be reversed as long as the functions of the chloroplasts can be restored (Van Doorn and Yoshimoto, 2010). We wondered whether the capacity of a plant to control senescence could be somehow linked to catabolism of PAs. To verify this, we measured the photosynthetic quantum conversion parameters and leaf nitrogen (N) status in senescing barley leaves after the addition of DAO and PAO inhibitors (**Figure 6**). In the control leaves, the values of maximum quantum yield of PSII in the dark-adapted state *(Fv*/*Fm)* and photochemical quenching of *Chl (qP)* underwent a relatively small decrease until day 7 of senescence and then declined 0.8× and 0.5×, respectively. Accordingly, non-photochemical quenching of *Chl* fluorescence *(NPQ)* was at normally low values and did not change much until day 7, after which it rose 2.1× in comparison to day 0. At the same time, the *Chl* and *NBI* indexes lowered gradually from day 0 to day 7 and stabilized at values 2.4× and 3.7× lower than at day 0. The flavonoids ratio (*Flv)* value raised slowly during senescence. At day 10 it reached a level 1.2× higher than at day 0. The addition of DAO inhibitor (AG) enhanced the effect of senescence on the photosynthetic parameters, slightly lowering the values of *Fv*/*Fm, Chl, qP* and *NBI*, which generally drop during the process, and further increasing the values of *NPQ* and *Flv*, which increase in senescing leaves. In most cases, this effect was enhanced at the later stages of senescence (days 7 and 10). When AG was applied to leaves together with GABA, the photosynthetic activity parameters and the progress of the chlorophyll degradation process remained at the control level. The effect of applying the PAO inhibitor (G) was opposite to that of AG, and the senescence-associated changes in photosynthetic efficiency of chloroplasts were minimized. Again, this effect was most prominent at the last analyzed stage (day 10).

### Effect of PAO Inhibition on Hydrogen Peroxide Generation in Senescing Leaves

The physiological role of PAs can also result from their involvement in the plant signaling network. PA oxidation via oxidases leads to the generation of hydrogen peroxide (H_2_O_2_), which is involved in stress responses and the induction of PCD (Moschou et al., 2008). The obvious effect of PAO inhibition of senescence-associated changes in cell metabolism encouraged us to analyze the senescence-related changes in H_2_O_2_ accumulation and the effect of inhibiting oxidase activity.

**FIGURE 7:**
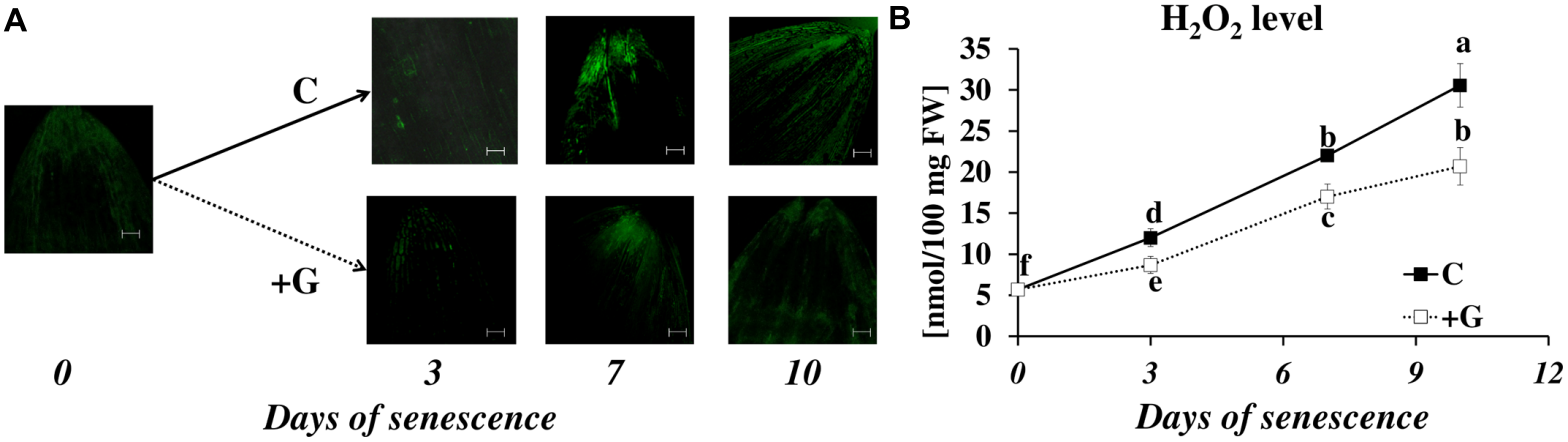
**Senescence-related hydrogen peroxide production in dark-incubation in the presence of PAO activity inhibitor in barley leaves. (A)** *In situ* detection of hydrogen peroxide with confocal microscopy and the fluorescent probe 2′,7′-dichlorofluorescein. The bar indicates 200 μm. **(B)** H_2_O_2_ level determined with a spectrophotometric method based on titanium (Ti^4+^) estimated throughout senescence in C (solid line)- control leaves and after application of polyamine oxidase (PAO) activity inhibitor, +G (dotted line) leaves treated with 50 μM guazatine (G) – PAO activity inhibitor. The differences in the parameters were analyzed for statistical significance using one-way ANOVA and the Tukey–Kramer Multiple Comparison Test (*n* = 9, *p* < 0.01). The same letters on bars describe non-significant differences between means.

In control plants, the DCFH-DA fluorescence intensity, which reflects H_2_O_2_ accumulation, increased as leaf senescence progressed (**Figure 7A**). The fluorescence was detected primarily in the external layers of the leaf tip, in the epidermis and the midribs. A weak signal was observed in the mesophyll cells, and a very weak signal in the veins and blades of leaves. The same trend was observed in plants subjected to inhibitor treatment, but their effect on the overall level of H_2_O_2_ production was the opposite. Inhibiting PAO activity lowered the fluorescence signal at days 3 to 10. The results of studies using confocal microscopy were also confirmed by the spectrophotometric studies (**Figure 7B**). The exposure of the leaf to dark conditions resulted in a continuous increase in H_2_O_2_ content at days 3, 7, and 10 (2.2×, 3.9×, and 5.3×, respectively, compared to the control). In leaves incubated with a PAO inhibitor, H_2_O_2_ content also increased but reached only 73% (day 3), 77% (day 7), and 68% (day 10) of the control level.

## Discussion

**FIGURE 8:**
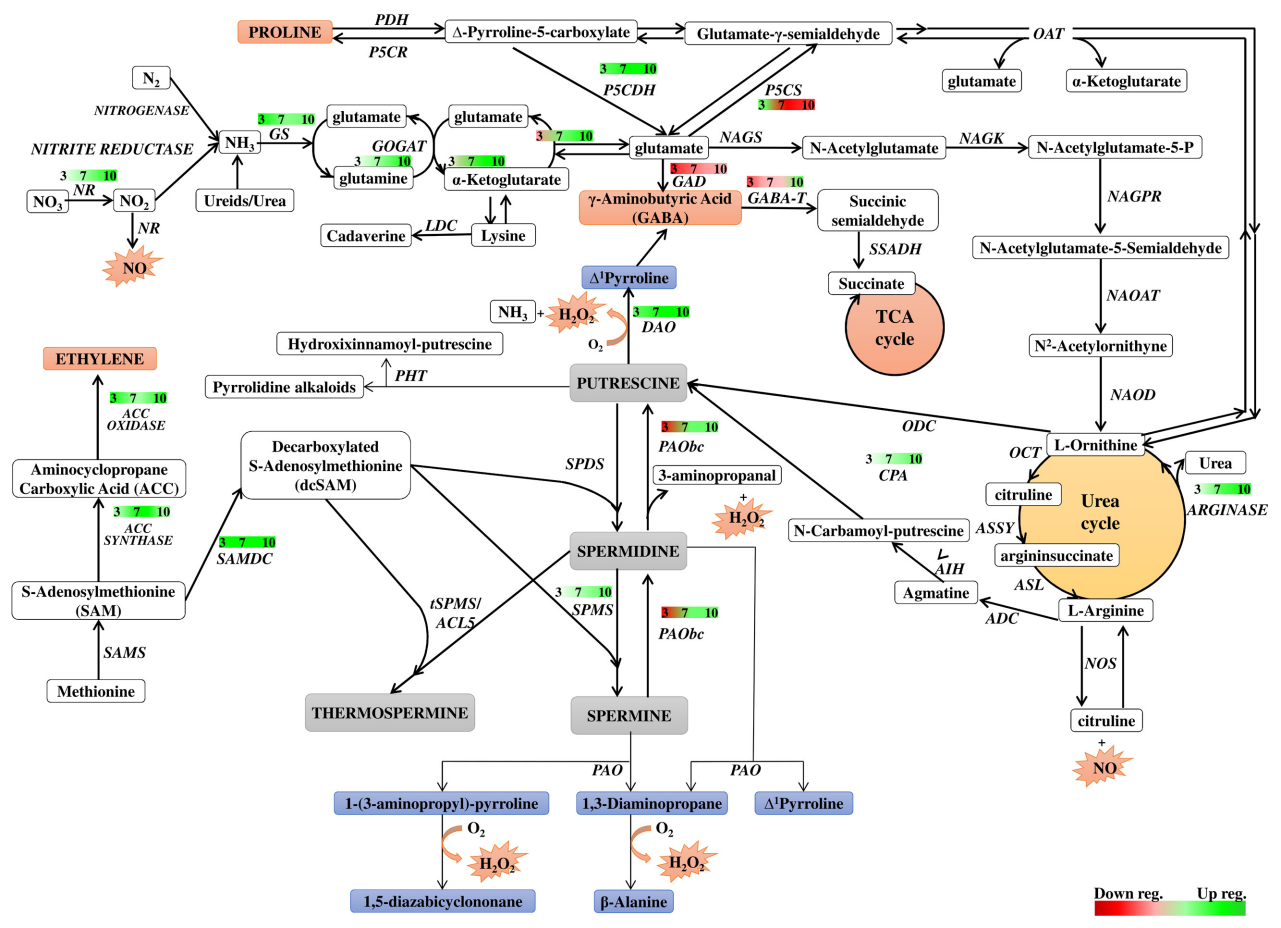
**Schematic presentation of possible interaction between polyamine metabolism and other pathways through dark-induced barley leaf senescence**. Polyamine biosynthetic routes are indicated with bolded lines. Thin lines mark putrescine-derived alkaloid formation, polyamine conjugation and catabolic processes. The red-green bars indicate the expression level of genes estimated according to the results of microarray analysis. Numbers in the bars describe the senescence days. NR, nitrate reductase; GS, glutamine synthetase; GOGAT, glutamine oxoglutarate aminotransferase; NAGS, *N*-acetylglutamate synthase; NAGK, *N*-acetylglutamate kinase; NAGPR, *N*-acetylglutamate 5-phosphate reductase; NAOAT, *N*-acetylornithine transaminase; NAOD, *N*^2^-acetylornithine deacetylase; OCT, ornithine carbamoyltransferase; ASSY, argininosuccinate synthase; ASL, argininosuccinate lyase; ADC, arginine decarboxylase; AIH, agmatine iminohydrolase; CPA, *N*-carbamoylputrescine amidohydrolase; ODC, ornithine decarboxylase; SAMS, SAM synthetase; SAMDC, SAM decarboxylase; SPDS, spermidine synthase; SPMS, spermine synthase; tSPMS/ACL5, thermospermine synthase/acaulis5; PAO, polyamine oxidase; PAObc, back conversion polyamine oxidase; SSAT, spermidine/spermine *N*-1 acetyl transferase; DAO, diamine oxidase; GABA-T, γ-aminobutyrate aminotransferase; SSADH, succinic semialdehyde dehydrogenase; GAD, glutamate decarboxylase; PHT, putrescine hydroxycinnamoyl transferase; LDC, lysine decarboxylase; NOS, nitric oxide synthase; P5CS, Δ^1^ pyrroline-5-carboxylate synthetase; P5CR, Δ^1^ pyrroline-5-carboxylate reductase; PDH, proline dehydrogenase; P5CDH, Δ^1^ pyrroline-5-carboxylate dehydrogenase; OAT, ornithine-δ-aminotransferase.

Polyamine metabolism is linked to many metabolic pathways in the cell by being involved in the formation of signaling molecules and metabolites directly related to the cellular response to environmental changes. The network of these relationships is shown in **Figure 8**, which is useful background to follow the interpretation of the results discussed below. **Figure 8** also summarizes the time course of expression of genes involved in or related to PA metabolism in dark-induced barley leaf senescence.

Our study revealed a senescence-dependent interplay between various PA forms and individual PAs. At the beginning of the senescence process, a rapid increase in the level of all free PAs was observed, but at day 7, PU, SD, and SM levels began to drop to or even below the control level, while the DP level continuously increased.

In the cell, the PA titer is carefully controlled (Cogen, 1998; Moschou et al., 2012; Moschou and Roubelakis-Angelakis, 2013, and references therein). PA biosynthesis, catabolism, conjugation, interconversion, and transport contribute to PA homeostasis (Angelini et al., 2010; Moschou and Roubelakis-Angelakis, 2013). Thus, the increase in the free PA titer at the beginning of senescence can constitute a part of a signal that leads the cell to process induction, or it might maintain its progress. As our study revealed, the PA accumulation upon senescence could be due to simultaneous up-regulation of a set of genes involved in PA biosynthesis and an increase in enzymatic activity of the proteins they encode. Senescence is sensitive to hormonal perturbation, with particular reference to cytokinins (Lim et al., 2007). Cytokinins prevent chloroplast degradation by retarding both the breakdown of chlorophyll and the decay of the thylakoid system in senescing leaves. They also lower the activity of proteolytic enzymes and promote protein synthesis in detached barley leaves senescing in the dark (Legocka and Szweykowska, 1981, 1983). The delay of senescence by cytokinins occurs mainly at the transcriptional level, by inhibiting the expression of senescence-related genes (Lim et al., 2007). We applied KIN to the model in this study to learn whether PA accumulation is an integral part of senescence–dependent events, as well as whether PAs might act as drivers or controllers of the process. We showed that in the presence of KIN, the PA accumulation shifted to later times, confirming that it is a senescence-dependent. It also implied that some additional senescence-related signals, blocked by exogenously applied KIN, influence PA synthesis in this process.

When studying dark-induced PA accumulation, it is important to note that induced PA accumulation at the beginning of the process could imply a ROS-scavenging function of both free and conjugated forms of PAs, as reported by Radyukina et al. (2009) and Legocka et al. (2015). SD and SM, which have three and four amino groups, are more effective in the removal of reactive oxygen forms than PU (Kuznetsov and Shevyakova, 2007).

We also measured transcript levels and enzymatic activity of the PA catabolic enzymes DAO and PAO, as well as the effect of their inhibition. Our data indicated that transformations between individual PAs could be an essential element of darkness-induced responses and indicate the physiological role of PA in cell senescence. Inhibiting PAO activity drastically slowed down the accumulation of both DP and PU, while the levels of SD and SM were substantially increased. This is a logical effect, but remarkably also resulted in slowing down the senescence-associated chlorophyll loss. Application of PAO inhibitors maintained the *Chl* parameter at a higher level in leaves subjected to senescence compared to those incubated in the absence of inhibitor, which also resulted in higher values of the chlorophyll fluorescence parameters such as *Fv*/*Fm* and *qP*. Furthermore, inhibiting PAO activity decreased the amount of H_2_O_2_, suggesting that PAO-mediated catabolism of SD/SM supports senescence-dependent degradation, most likely by H_2_O_2_ synthesis. Plant responses to environmental factors involve the secretion of SD/SM to the apoplast where they are catabolized, leading to H_2_O_2_ production. Depending on the amount of H_2_O_2_, the defense response or cell death program is initiated (Yoda et al., 2003, 2006; Marina et al., 2008; Moschou et al., 2008). In agreement with this model, the high SM/SD pool in the apoplast rapidly increased at day 3 and was maintained at a high level, which corresponded with the gradual accumulation of DP and the increase in H_2_O_2_ level.

The initial amount of PU in the apoplastic pool of PAs was one order of magnitude lower and only slightly increased during senescence. However, PU dominated in the free PA fraction, where it initially accumulated to a high level, and then started to drop after day 3. This effect was accompanied by the formation of PU conjugates that accumulated in the senescing leaf to a high level, indicating the fate of PU in the senescing cell. For many years, PA conjugates were considered to be inactive PA forms. However, recent evidence indicates that they are essential for certain developmental processes (Bassard et al., 2010; Fuell et al., 2010). Senescence-dependent nitrogen (N) and carbon (C) flow might be then shifted toward PA conjugation, which could explain the observed increase in expression of respective protein coding genes. The hypothesis that PAs are significant players in the turnover of N molecules in plants has been discussed by Mattoo et al. (2010). Furthermore, our results strongly indicate the involvement of a DAO-mediated PU oxidation process in GABA production. Microarray-based profiling of GAD gene expression suggested that in senescing leaves, GABA synthesis from glutamate is gradually suppressed. PU oxidation might become an alternative source of GABA to the tricarboxylic acid cycle and also for some as yet undefined signaling pathways. The role of GABA as a neurotransmitter is well-established in animal cells. In plants, increases in the GABA level have been reported in response to different stresses. However, the exact role of GABA under stress conditions still needs to be defined (Michaeli and Fromm, 2015). Analysis of *Chl a* fluorescence parameters and plant N status revealed that blocking the PU oxidation pathway accelerated chlorophyll degradation. However, simultaneous addition of exogenous GABA together with a DAO inhibitor was sufficient to prevent accelerated degradation of photosystem complexes. Together, these results point to a central role of GABA signaling in senescence and indicate that PU may be a key precursor of this transmitter.

## Conclusion

The results demonstrate that the dark-induced leaf senescence process was supported by the accumulation of SD and SM. Both were newly produced and transported into the apoplast, where they were readily degraded, yielding hydrogen peroxide and DP, which could regulate or participate in senescence-dependent degradation processes. However, PU also accumulated in the senescing leaf. As indicated in the experiment with cytokinin, the PA accumulation was senescence-dependent, although PA anabolism could not promote or abrogate the process started by dark incubation in the absence of additional senescence-related signals. Thus, the accumulation of PU might serve as a precursor of SD and SM but also support the senescence process through forming PU conjugates, the only PA conjugates forming in senescence, and by supporting via its oxidation GABA synthesis.

The knowledge of the participation of PAs in leaf senescence is very fragmentary. Our results reported here shed some light on the problem, in particular on leaf senescence in an important crop. These insights allow the development of a framework that would provide more detailed observations into induced senescence and its biotechnological applications and would stimulate new, important questions about the function of PAs in the process.

## Author Contributions

ES-N, JL conceived and designed the experiments. ES-N, SK, AZ, AM performed the experiments. ES-N, SK, AZ, AM analyzed the data. ES-N, AZ, AM contributed reagents/materials/analysis tools. ES-N, AZ wrote the paper. ES-N, SK, AZ, AM, JL participated in manuscript finalization and preparation of Figures.

## Conflict of Interest Statement

The authors declare that the research was conducted in the absence of any commercial or financial relationships that could be construed as a potential conflict of interest.

## ABBREVIATIONS

ADC: arginine decarboxylase
AG: *N,N*-diaminoguanidine
CPA: *N*carbamoylputrescine amidohydrolase
DAO: diamine oxidase
DP: diaminopropane
G: guazatine
GABA: γ-aminobutyric acid
GABA-T: γ-aminobutyric aminotransferase
GAD: glutamate decarboxylase
GDH: glutamate dehydrogenase
GOGAT: glutamine oxoglutarate aminotransferase
GS: glutamine synthetase
KIN: kinetin
NR: nitrate reductase
PAO: polyamine oxidase
PAObc: back conversion polyamine oxidase
PAs: polyamines
PCD: programmed cell death
PU: putrescine
P5CS: Δ^1^ pyrroline-5-carboxylate synthetase
P5CDH: Δ^1^ pyrroline-5-carboxylate dehydrogenase
RuBisCO: ribulose-bis-phosphate carboxylase
SAMDC: *S*-adenosylmethionine decarboxylase
SD: spermidine
SM: spermine
SM/T: thermospermine
SPMS: spermine synthase.
